# Coordination patterns reveal online political astroturfing across the world

**DOI:** 10.1038/s41598-022-08404-9

**Published:** 2022-03-17

**Authors:** David Schoch, Franziska B. Keller, Sebastian Stier, JungHwan Yang

**Affiliations:** 1 GESIS – Leibniz Institute for the Social Sciences, Cologne, Germany; 2 University of Bern, Bern, Switzerland; 3 University of Illinois at Urbana-Champaign, Champaign, USA

**Keywords:** Information technology, Computational science

## Abstract

Online political astroturfing—hidden information campaigns in which a political actor mimics genuine citizen behavior by incentivizing agents to spread information online—has become prevalent on social media. Such inauthentic information campaigns threaten to undermine the Internet’s promise to more equitable participation in public debates. We argue that the logic of social behavior within the campaign bureaucracy and principal–agent problems lead to detectable activity patterns among the campaign’s social media accounts. Our analysis uses a network-based methodology to identify such coordination patterns in all campaigns contained in the largest publicly available database on astroturfing published by Twitter. On average, 74% of the involved accounts in each campaign engaged in a simple form of coordination that we call co-tweeting and co-retweeting. Comparing the astroturfing accounts to various systematically constructed comparison samples, we show that the same behavior is negligible among the accounts of regular users that the campaigns try to mimic. As its main substantive contribution, the paper demonstrates that online political astroturfing consistently leaves similar traces of coordination, even across diverse political and country contexts and different time periods. The presented methodology is a reliable first step for detecting astroturfing campaigns.

## Introduction

At the very latest since the Russian Internet Research Agency’s (IRA) intervention in the U.S. presidential election 2016, scholars and the broader public have become wary of online disinformation campaigns^1^. Such campaigns often aim to deteriorate the public’s trust in electoral institutions or the government’s legitimacy – or try to shore up support for authoritarian governments. Some are successful in that regard, as experimental research has demonstrated^2,3^. While there is disagreement in large-scale observational studies regarding the prevalence of untrustworthy online information in citizens’ information diets^4–6^, there is a consensus that disinformation is a major problem, whether it emerges organically from communities or is pushed by systematic campaigns.

However, current scholarship on disinformation campaigns is largely focused on the detection of automated accounts, so-called social bots^7–9^, even though it has been shown that such accounts make up only a small part of contemporary astroturfing campaigns^10^ and the validity of the bot-detection methods is in question^11^. To fill this research gap, our study focuses on “political astroturfing”, i.e., centrally coordinated disinformation campaigns in which participants pretend to be ordinary citizens who act independently^12^. The accounts that are associated with political astroturfing may or may not be automated social bots and may or may not spread “fake news”^13^, misinformation, or disinformation^14–18^ but they do deceive the audience by disguising their identity and hiding the motivations of the account owner.

We argue that these campaigns can be more accurately detected by searching for *centralized coordination patterns among groups of accounts* instead of looking at *“suspicious” activity of individual accounts*. After all, political astroturfing is an organized campaign activity that entails a coordination of multiple accounts. Our past research on astroturfing by the South Korean secret service in 2021 demonstrated that the central coordination and organizational routines inherent to an information campaign allows researchers to distinguish between campaign agents and ordinary Twitter users^10,19^. Similarly, recent studies examined coordination patterns of the accounts that were harassing members of the Iranian diaspora on Instagram^20^ and hijacking German Twitter debates during an election campaign^21^. To examine whether the relatively simple coordination patterns described in previous research can be used to detect a wide variety of astroturfing campaigns in more recent years, we apply the method developed in Keller et al.^10^ to all astroturfing campaigns revealed by Twitter. We then demonstrate the generalizability of the detection method by testing its performance on more recent campaigns that engaged in different strategies, targeted different audiences, and used different languages. Overall, we argue that identifying and restricting astroturfing campaigns is important for leveling the playing field for political debates in democratic societies.

## Theory and research approach

Our central argument is that studying the timing and centralization of message coordination helps locate the activity of a set of accounts on an empirically observable spectrum of group-based behaviors on social media. Such a spectrum ranges from “uncoordinated messages of unrelated users” to “centrally coordinated information campaign”. Astroturfing – in particular if campaigns employ unsophisticated social bots – will be placed on the latter end of the spectrum that exhibits a strong group-based coordination. One theoretically relevant question is the location of grassroots movements in this space. Grassroots movements exhibit some coordination but are less synchronized in the timing and content of their messages, because their participants respond organically to cues sent by their peers instead of centralized instructions. Therefore, they will appear in the middle of the spectrum. As a consequence, the centrally coordinated organization of astroturfing should leave different empirical traces than grassroots campaigns.

We ground our explanation of these patterns in social science theory, more specifically, the principal–agent framework as employed in political science^22^, where it has been used to explain the pitfalls of ground campaign organization during elections^23^. Applying the framework to astroturfing, we argue that the organizers of an astroturfing campaign are *principals* who try to pursue (political) goals by instructing and incentivizing *agents* to create and share messages congruent with the campaign’s goals. Because one of the purposes of astroturfing is to reach and change the behavior of as many *regular users* as possible, the success of the campaign is contingent on a wide reach and an organic appearance of the campaign. However, according to principal–agent theory, reaching this more complex goal is difficult because of the misalignment between the principal’s and the agents’ preferences: agents thus need to be extrinsically motivated and will try to shirk^22^, e.g. by creating similar or identical accounts and content instead of coming up with original contributions to the campaign. Unless the principal can establish an expensive system of close monitoring, the agents will continue to hold an information advantage over the principal: they know how much effort they exerted in creating convincing online personas (oftentimes, little), whereas the principal does not.

We thus would expect campaign accounts to post or re-post similar or identical messages within a short time window, something we call *co-tweeting* and *co-retweeting*, and coordinate with a large number of other campaign accounts. This might be similar to grassroots campaigns’ operations, but their activities do not follow centralized instructions, and are therefore more likely to post similar content in a cascading fashion over an extended time period, and with greater variation in the content as they engage in more localized coordination with a few friends, imitating and varying the content they see online. Finally, grassroots campaigns may rely heavily on Twitter’s decentralized mechanism for spreading the message, *retweeting*. But as astroturfing campaigns use retweeting as well^10^, this is unlikely to be a useful feature to distinguish the two. Finally, principal–agent theory predicts that agents will only extend their efforts when they are supervised, which might result in *unique temporal activity patterns*, e.g., posting only during regular office hours. We expect that this principal–agent constellation results in universal patterns that appear in astroturfing campaigns in multiple countries across the world.

**Figure 1:**
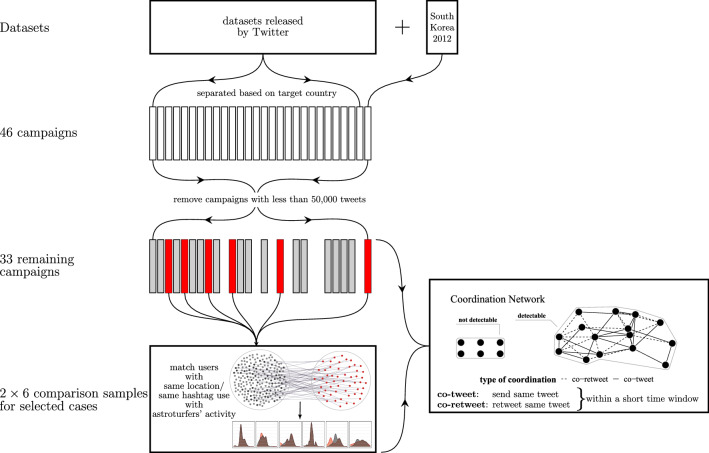
Research design. Datasets, case selection and analysis approach.

Figure 1 shows a schematic representation of our research design. We use the complete data released by Twitter as part of its *Information Operations Hub* initiative^24^ up until February 2021 as “ground truth” data to show that similar coordination patterns appear in almost all cases. We also include a campaign that escaped Twitter’s attention, namely the South Korean secret service’s attempt at influencing the national elections in 2012, bringing our total population of astroturfing campaigns to 46. We then concentrate the focus of the study on the 33 campaigns that produced at least 50,000 tweets. In order to validate methods for the detection of astroturfing, we compare the behavior of astroturfing accounts to two “comparison samples” that represent groups of users that a given campaign likely tries to mimic and influence. As retreiving the tweets for such systematic samples is time and resource intensive, we select four distinct campaigns targeting audiences in six countries for an in-depth study: the Russian Internet Research Agency’s (IRA) attempt at polarizing public opinion in the U.S. and Germany, and shoring up regime support in Russia, the Chinese government’s attempt at changing the framing of the Hong Kong protest, a campaign cheerleading the government of Venezuela and the above mentioned South Korean case. Further details on case selection and sample construction can be found in the Methods section.

Defining adequate comparison groups is essential in this detection process because (decentralized) coordination can also happen as part of organic discussions among specific issue publics and grassroots movements. To demonstrate the added value of our research in that regard, we conducted a literature review of related research aiming to detect astroturfing (see Table S1 in the Supplementary Information [SI]). We excluded (1) studies that aim to detect social bots, as this research does not aim to reveal specific astroturfing campaigns but general automation patterns instead, and (2) studies that merely use the data released by Twitter for an analysis of astroturfing without aiming to predict which accounts are part of a specific campaign. The latter category includes a number of studies that examined the character and reach of individual astroturfing campaigns in various contexts such as China^25^, Saudia Arabia^26^ or the Russian influence campaign in the U.S.^27–30^).

Among the research listed in Table S1^10,31–34^, the studies most closely related to ours are Vargas et al.^34^ and Alizadeh et al.^31^, both of which use some of the same data to distinguish between astroturfing accounts and regular users. In particular, Vargas and colleagues built on our earlier work^10,19^ with the goal of constructing a classifier to detect astroturfing campaigns more generally^34^. However, they chose baselines that represent neither genuine grassroots movements nor the specific issue publics targeted by the campaigns, but instead three institutionalized English-speaking elite communities (members of the U.S. Congress, the UK Parliament and academics). Not only do these communities differ from the country-specific audiences engaged in the topics targeted by an astroturfing campaign, but the U.S. and UK parliamentarians’ accounts are often run by a group of staffers who work for the politicians. These political elites’ accounts are therefore unlikely to act like an account owned by an ordinary citizen. As a second baseline, they used a snowball sample of random users without any relation to the conversations in the target countries at the time the astroturfing campaigns were active.

The paper by Alizadeh et al. focusing on English-language astroturfing campaigns uses a subset of the Twitter data to build an algorithm that can detect other astroturfing accounts in later time periods, on other platforms, or later campaigns initiated by the same actor^31^. In other words: their approach requires a set of astroturfing accounts already identified using alternative methods. They compare the known astroturfing accounts to a random sample of regular and politically interested users. While the latter may seem like an appropriate comparison group, the authors define politically interested users as those users who follow at least three politicians – the sample therefore may contain bots designed to boost follower counts and does not consist of accounts that engage in the debates that the astroturfing campaign tries to influence.

We construct more natural comparison samples that reflect specific contexts of each astroturfing campaign, such as country and campaign-related topics and keywords. We randomly sampled users located in the targeted country that engage in the discussions that the astroturfing campaigns try to influence to compare their activity patterns with those of astroturfing. We also take the level of activities into consideration when we design comparison samples, such that the accounts in the astroturfing campaigns and the comparison groups are comparable. Taken together, our paper presents a scalable method for detecting astroturfing campaigns and validates the findings against the very accounts the campaign tries to mimic. This universal approach does not require any training data and performs well without human inputs when detecting groups of suspicious accounts in all previously revealed instances of astroturfing on Twitter. With that, the study contributes to methodological approaches for the detection of disinformation and reveals surprising similarities in astroturfing campaigns, even across heterogeneous political and social contexts.

## Results

**Figure 2:**
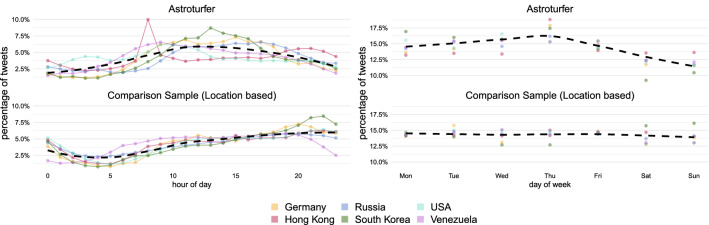
Astroturfing campaigns are most active during office hours and working days. Comparison of hourly (left) and weekly (right) activity of astroturfing campaigns and location-based case-specific random samples. Dashed line indicates the average activity.

Journalistic investigations of astroturfing campaigns indicate that campaign participants are often hired and work in shifts (see, e.g. the New York Time’s reporting on the Russian trolls^35^) or during regular office hours. This might result in activity patterns that are consistent across all campaigns, but distinct from those of regular users. Figure 2 shows that a large fraction of the astroturfing campaign tweets (top) are posted during regular office hours, and not in the evening, when regular users (bottom) tend to be more active. Astroturfing tweets are also less likely to be posted during the day(s) off in the target country. These results hold irrespective of whether the comparison sample are regular users based in the target country, or users sampled because they – in addition – are part of the issue public the campaign tries to infiltrate, i.e. they use the same hashtags as the astroturfing campaign in the specific time window (see Methods section and additional evidence in SI S4). The pattern also generalizes across all datasets released by Twitter: activity drops on Saturday and Sunday in the case of countries with a Christian cultural background, or Friday in the case of campaigns associated with Muslim-majority countries (e.g., Iran and the UAE, see SI S4).

Not every account that is predominantly active during business hours is an astroturfing account. More telling in that regard are patterns indicating that a synchronous coordination is taking place. To descriptively chart traces of coordination, we use the heat maps of their daily activity. Just by ordering all accounts according to their level of activity (i.e., how many tweets they posted) in SI S5, it becomes clear that all astroturfing campaigns contain groups of accounts that increase or cease their activity at similar time points. This is another pattern related to astroturfing campaigns’ central coordination, in which participants receive simultaneous instructions to increase their activity or create new accounts, or due to shirking, i.e., the agents’ usage of desktop applications that allow for posting from several accounts simultaneously.

**Figure 3:**
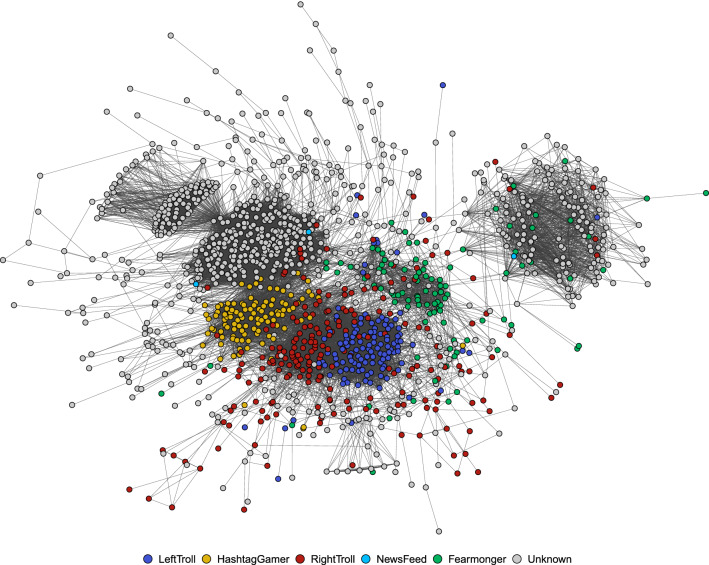
Astroturfing campaigns that target diverse audiences (here: Black Lives Matter activists in blue and Trump Republicans in red) are still connected in a message coordination network. Combined co-tweet and co-retweet network among IRA accounts that tweet in English. Node color is taken from a typology of Linvill and Warren, who manually classified IRA accounts according to their content^29^.

These patterns can be broken down to the level of individual tweets by creating networks based on message coordination, specifically if accounts tweet or retweet the exact same message within a short time window. We use such instances of “co-tweeting” and “co-retweeting” to link two accounts and create networks among the accounts examined (see section Methods for details). To illustrate the approach, Fig. 3 shows the combined co-tweet and co-retweet network for a more complex campaign that was targeting heterogeneous audiences. Even accounts belonging to the infamous IRA campaign in the U.S. that targeted both a right-wing (Trump supporters) and a left-wing (Black Lives Matter) audience^29^ still formed one large network component. A closer inspection of the content shared by both sides indicates that accounts pretending to be BLM activists and those posing as black gun supporters for instance found common grounds around the hashtag #OscarsSoWhite.

**Figure 4:**
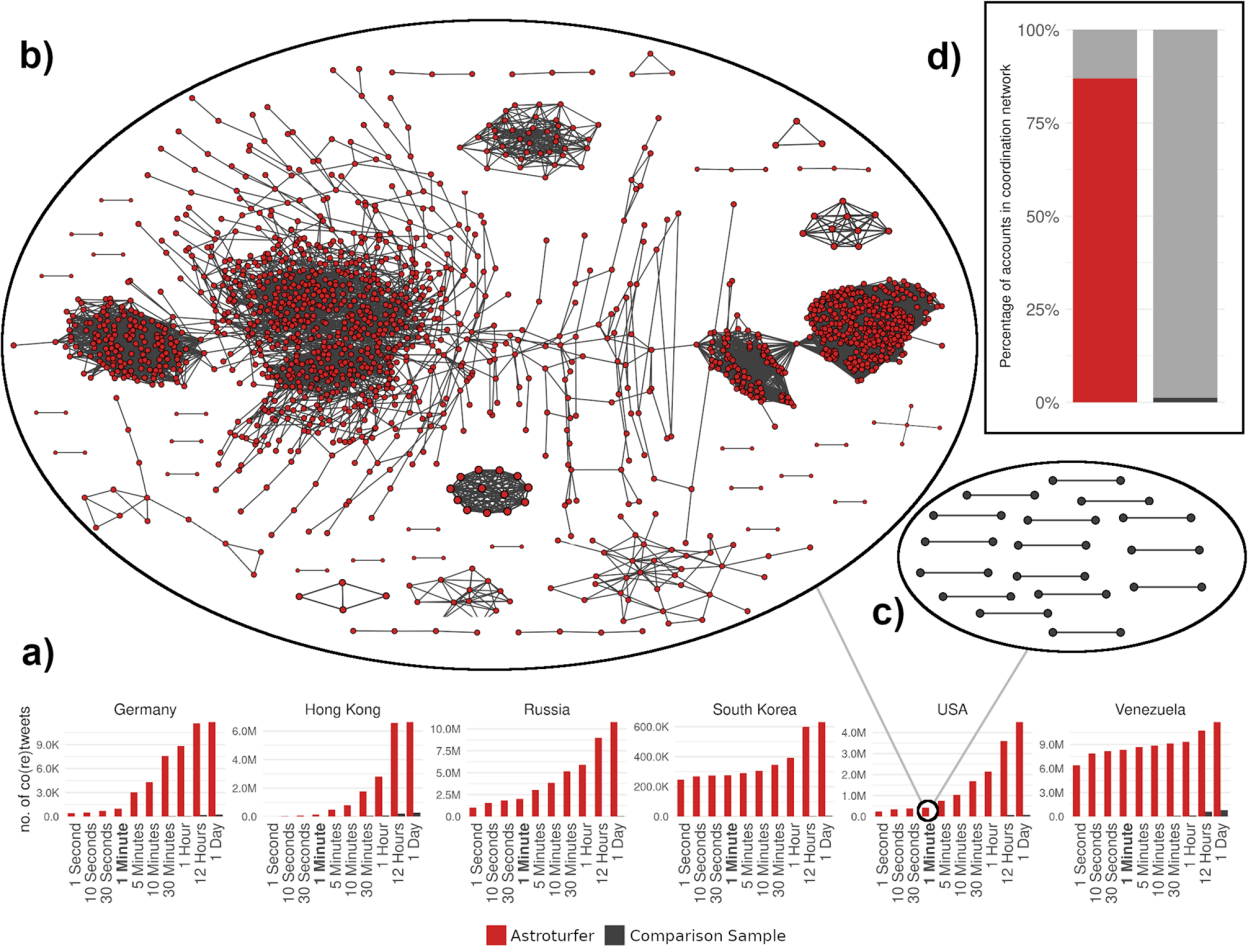
Message coordination in astroturfing campaigns (red) is more common and involves more accounts than in a comparison group of regular Twitter users (black). (**a**) Comparison of the number of co-(re)tweets among accounts with varying temporal threshold (i.e. how far apart two tweets are allowed to be in order to still be considered a co-(re)tweet. (**b**) Co-(re)tweet network of the U.S. campaign. Two accounts are connected if they (re)tweeted the same content within one minute. (**c**) Same as b) but for an equally sized set of comparison users located in the U.S. (**d**) Percentage of accounts (astroturfing or comparison group) appearing in the networks shown in (**b**) and (**c**). Missing accounts to 100% are isolated in the network, meaning that they do not co-(re)tweet with any other account in the respective sample.

Figure 4 displays the combined co-tweet and co-retweet network for the IRA campaign in the U.S. (b), and for its baseline group of comparison users (c), using our preferred temporal threshold, one minute. In that particular case and based on that particular network or decision rule alone, we would detect more than 80 per cent of the astroturfing accounts, while the number of false positives hovers around 1 per cent (d). As the Figs. in SI S6 show, the accounts involved form large connected network components in every single campaign, while regular accounts form no or rather small network components. Finally, the bar charts in panel (a) at the bottom of Fig. 4 demonstrate that the differences in message coordination between the astroturfing accounts and regular users remain large, irrespective of the time window used.

While there are at least three ways in which accounts could coordinate their messages – co-tweeting, co-retweeting and retweeting – we find that a combination of the first two most reliably detects centrally coordinated campaigns. Detecting accounts based on retweeting results in the highest number of false positives across the three measures, in particular in the Venezuelan random samples (see the full network Fig. in SI S6). This is not surprising, as retweeting is the most decentralized form of coordination: individual accounts can make an independent decision to share an interesting tweet and in that process participate in an issue public. While to a lesser degree this is also true for co-retweeting, repeated co-tweeting still seems implausible in the absence of (centralized) coordination in which accounts are being told what to tweet about or in which one actor controls multiple accounts. Another reason why it is preferable to use co-tweeting and co-retweeting is that detection based solely on retweeting requires prior knowledge about at least some campaign participants, which renders the method impractical for the real-time detection of astroturfing. Therefore, for summarizing the performance of our methodology, we rely on the co-tweet and co-retweet networks which can universally be constructed from any Twitter dataset.

**Figure 5:**
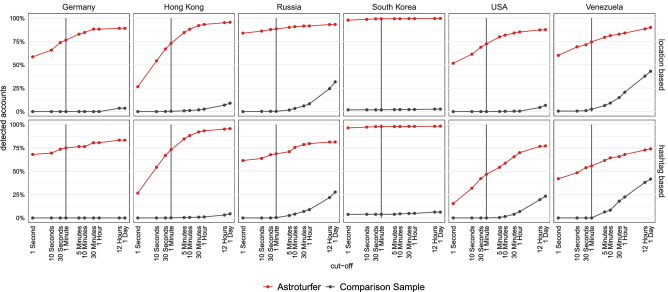
Astoturfing accounts are much more likely to coordinate their activity than comparison users for any given threshold. Astroturfing accounts (red) and comparison user accounts (black) classified as astroturfing accounts, location-based (top row) and hashtag-based (bottom row). Based on appearance in either the co-tweet or co-retweet network, depending on different temporal thresholds.

Figure 5 shows the percentage of detected astroturfing accounts for different time windows during which a pair of accounts either co-tweeted or co-retweeted together. That means an account is considered “detected” if it is not an isolate in either of the two networks. Just using the two metrics and a threshold of one minute allows for the identification of a high share of astroturfing accounts, while the false positive rate, i.e., the number of falsely flagged regular users, only increases when significantly relaxing the temporal threshold. The most reasonable threshold seems to be below 8 hours, i.e., less than the duration of a workday. This pattern is in line with media and insider reports suggesting that agents in astroturfing campaigns often receive instructions on a daily basis.

To add an organic grassroots campaign as an additional baseline, we collected data from a recent German (COVID-19 vaccination) information campaign, #allesindenArm as yet another benchmark sample. Under the hashtag #allesindenArm (roughly translated as “get a jab”) influencers and regular social media users posted personal messages to motivate other users to get vaccinated. There is no indication that this campaign was supported by astroturfing and we assess it as a genuine grassroots campaign. Despite some (open) coordination among participating users, the results in SI S7 show that the level of message coordination via co-retweeting and cotweeting is roughly equivalent to the random samples and therefore clearly distinguishable from the level of coordination in an astroturfing campaign. We also show that the temporal patterns of this genuine campaign (daily and hourly frequency of tweeting) resemble that of the accounts in the random samples.

**Figure 6:**
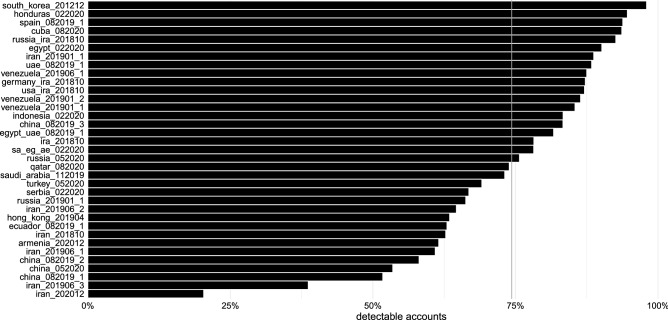
The methodology detects on average 74% of all accounts involved in a campaign. Detected astroturfing accounts based on appearance in either the co-tweet or co-retweet network (one-minute threshold) for all campaigns released by Twitter with more than 50,000 tweets. Campaign labels reflect information provided by Twitter in their data releases. Grey line indicates the mean.

After validating the approach against comparison user baselines, we finally apply the methodology to all astroturfing campaigns published by Twitter. Figure 6 shows that on average, 74% of astroturfing accounts were detected. The variation across campaigns cannot easily be explained by a clear geographical or geopolitical pattern. This indicates that specific campaigns differ in how well they navigate principal–agent problems.

## Conclusion

This paper presented the most comprehensive investigation of political astroturfing campaigns across the globe. Our analysis spanned various political and cultural contexts while covering multiple continents and time periods. Despite this heterogeneity, we found remarkably similar patterns in all astroturfing campaigns using the detection method by Keller et al.^10^. The findings suggest that astroturfing exhibits universal features that could help researchers, social media companies, and citizens to identify these types of disinformation. Our theoretical framework relates this to principal–agent theory: unlike the participants of genuine grassroots movements, astroturfing agents are not intrinsically motivated. Therefore many agents involved in astroturfing campaigns invest little time in creating distinctive online personas or varying their behavior across the accounts they control. Such patterns are difficult to camouflage, because message coordination is inherent to any information campaign, and resources to mitigate principal–agent problems are usually limited.

In contrast to much previous literature, our methodological approach detects astroturfing campaigns based on the patterns of coordinated group efforts to produce messages instead of focusing on individual account features, such as heavy automation. By relying on a parsimonious set of metrics, we also provide a transparent methodology that can be universally applied. We argue that this is an improvement over machine learning classifiers that might achieve a better performance as they are typically trained on a large number of case-specific features, but fail when trying to identify out-of-sample accounts^34^. For six cases, we constructed comparison groups of users that were engaging in salient political conversations in each of the target countries while the astroturfing campaigns were unfolding. It is important to underscore that our comparison groups were based on an account-level data collection aimed to find comparable accounts that produced tweets at similar rates with the astroturfing accounts. These comparisons consistently show that unique coordination patterns are evident only among astroturfers.

One limitation of our validation procedure is that Twitter might have used similar techniques for detecting astroturfers in the first place. To avoid circumvention, Twitter does not reveal the exact methodology used to detect the campaigns it removes from the platform. In statements accompanying the data releases, Twitter mentions information provided by industry partners and even law enforcement, as well as account-based features only the company has access to, such as IP addresses. We also note that data on the Iranian campaign was released in batches several months apart, even though the accounts contained in it engaged in co-tweeting across those batches. This leads us to believe that Twitter may (at least at that time) not have relied on too similar an approach as we propose. To safeguard against this potential endogeneity, our analysis also relied on data from a South Korean astroturfing campaign, where account names were directly retrieved from laptops of secret service agents.

The major advantage of the methodology is that it can be applied to almost any social media platform – as long as the platform allows users to post content (and therefore two accounts to post the same content within a certain time window) and to share content posted by other users (and therefore allow two accounts to simultaneously share it). Although this study did not analyze data from other social media platforms due to various constraints in data acquisition, it is crucial that researchers investigate which patterns universally apply to astroturfing across multiple social media platforms to further advance the findings of our study.

## Methods

The study was approved by the Human Participants Research Panel at the Hong Kong University of Science and Technology (G-HKUST601/19, HPR #382).

### Data

Starting in October 2018, Twitter has released data sets of tweets by accounts that it deemed to have been involved in hidden information campaigns. These campaigns occurred across different continents over the last decade, targeted different audiences domestic and abroad, and were conducted in different languages (the full overview is in SI S2). Since constructing meaningful baselines is resource-consuming, we chose cases to cover a wide range of campaigns, after excluding campaigns with less than 50,000 tweets.

Some campaigns targeted one language community only, while others targeted different language communities in different countries, such as the Iranian or the IRA campaign. The campaigns also had, judging from journalistic accounts thereof, different goals: the IRA campaign in the USA and Germany targeted at least two different communities on opposite ends of the political spectrum, while the campaign targeting its own country had the single focus of applauding the government. The Chinese campaign targeting Hong Kong had a similarly unified message: to vilify the protesters and some other perceived enemies of the PRC. For our analysis, we therefore selected the IRA campaign and split it into three different campaigns based on the three most prevalent languages: one targeting the US and its election, one Russia itself, and one targeting Germany. There are also multi-lingual campaigns that target a polyglot audience: we picked the example of the Chinese campaign influencing the Mandarin/Cantonese/English-speaking audience abroad and in Hong Kong. Among the mono-lingual campaigns, we selected the Venezuelan campaign to also include South America in our global coverage. We also examine a set of astroturfing accounts that was not revealed by Twitter, but in South Korean court documents^10^.

While our detailed analysis requires comparison data from a systematically constructed set of random users, we show in SI S4 that the descriptive temporal patterns are similar in all campaigns revealed by Twitter.

### Network analysis

We build on a methodology for the detection of astroturfing that exploits the principal–agent problems of such campaigns^10^. The behavioral patterns caused by strategic coordination are difficult to mask; therefore, they can be used as general indicators to find the agents associated with a broader disinformation campaign.

We distinguish three different measures of coordination patterns^10^. The most well-known form of coordination is *retweeting*: a large proportion of the retweets posted by astroturfing accounts tends to come from other campaign accounts. But as retweeting is also common among genuine grassroots campaigns, this measure creates the largest number of false positives, misidentifying potentially genuine grassroots movements as astroturfing, or including genuine converts of the campaign. A second form of coordination is *co-tweeting*, the act of two accounts posting the same message within a short (here, one minute) time window. This type of coordination is most likely to distinguish regular users from astroturfing accounts, as the former rarely post the same original message at the same time. But astroturfing accounts that exclusively amplify messages via retweeting will not get captured with this method. In order to capture this common type of behavior, we use *co-retweeting networks*: when two accounts retweet the same message within a one minute window, we construct a tie between them – but only if this occurs at least ten times. This behavior should be particularly widespread in campaigns that focus on boosting the visibility of their own and third-party accounts with little effort in creating original content.

We chose thresholds and time windows based on previous research where it became apparent that these can be varied without changing the main results^10^. Robustness tests with different temporal thresholds can be found in SI S5. In all campaigns, we prune the set of accounts to only include accounts that tweet more than ten times in the whole time period.

### Sampling user accounts for comparison

Finding the right criteria for a baseline to compare the campaign accounts to is not easy. Our comparison groups are two conditional random samples of Twitter users designed to look as similar to the group of users that the astroturfing campaign tries to mimic, making the distinction between astroturfing and regular users as challenging as possible. A random sample of all Twitter users, is likely to yield trivial results: a group of accounts from different parts of the world tweeting in different languages and different time zones is unlikely to tweet many similar messages at the same time. In addition, Twitter activity is distinctly right-skewed, and a random selection of accounts will yield a large number of inactive or barely active accounts. Accounts that do not tweet of course also do not coordinate their messages.

We therefore argue that the most appropriate comparison group is a set of accounts sampled from the users that the astroturfing campaign tries to emulate, stratified by level of activity. We collected our data from the commercial social media monitoring company Brandwatch. Brandwatch infers the country of a Twitter account based on geo-coordinates of tweets, the self-reported location information in user profiles, the time zone defined by users, top-level domains and geo-IPs of the links shared by a user as well as the language in tweets. Using geo-located tweets as benchmark, the company developed algorithms able to assign 90% of non-geolocated tweets to a country with that procedure. We start by drawing a random sample of tweets posted by accounts that Brandwatch’s algorithm locates in the targeted country (i.e., in the case of the IRA’s 2016 U.S. Presidential election campaign, users located in the U.S.). We then collected a random sample of the accounts appearing in this sample, collected all their tweets in the relevant time period and calculated their activity level (i.e., number of tweets posted). We matched each astroturfing account by activity level with an account from this random sample (see SI S2 for an evaluation of the matching procedure). We call this comparison sample the *location-based comparison sample*.

However, this procedure may still yield a group of random accounts so dissimilar that the exercise of distinguishing them from the campaign accounts is trivial. They could, for instance, be similarly active sports or pop music fans. This is why Alizadeh et al. use politically interested users as a comparison group, and identify those users by whether they follow known political figures^31^. But such politically interested users can hold a variety of political views and participate in many different debates. We therefore argue that a more rigorous test for any detection method is a sampling based on members of “issue publics”: for each campaign, we identify at least one period in which it used a handful of hashtags with increased frequency for at least a few days. We take this as evidence that the campaign tried to infiltrate a specific debate and the community forming around it. We then collect a random sample of tweets also mentioning these hashtags during the same time frame – in addition to also being based in the appropriate geographic location – and select random users from that sample to match the astroturfing accounts’ activity. We call this sample the *hashtag-based comparison sample*. This is a more appropriate comparison group than communities already existing within an institutional setting, such as members of the U.S. Congress, the British Parliament or academics^34^. After all, an astroturfing campaign does not pretend to be a well-established community, but regular users who happen to suddenly take interest in the same topic. SI S3 shows that the matching by activity was successful for each case and provides an overview of the samples.

## Supplementary Information

Supplementary Information.

## Data Availability

Data files necessary to replicate the results in this article are available on OSF: https://osf.io/ms5ue/?view_only=b020f97d49fc41b893391b0aef1bbfba.

## Code availability

R scripts that replicate the results in this article are available on OSF: https://osf.io/ms5ue/?view_only=b020f97d49fc41b893391b0aef1bbfba.
