# Supplementary material for: Coordination patterns reveal online political astroturfing across the world
Source: Sci Rep. 2022 Mar 17;12:4572. doi: 10.1038/s41598-022-08404-9 (PMC8930979; doi:10.1038/s41598-022-08404-9)
Supplement: Supplementary file 1 — Supplementary Information. [file 41598_2022_8404_MOESM1_ESM.pdf]

Coordination patterns reveal online political astroturfing across the world

David Schoch[1] Franziska B. Keller[2] Sebastian Stier[3]
JungHwan Yang[4]

[1]The University of Manchester
[2]Hong Kong University of Science and Technology
[3]GESIS – Leibniz Institute for the Social Sciences, Cologne
[4]University of Illinois at Urbana-Champaign

Supplementary Information

Replication materials are available on OSF:
https://osf.io/ms5ue/?view_only=b020f97d49fc41b893391b0aef1bbfba

S1 Related literature

Table S1: Research design of related studies

Study	Data	Astroturfing campaigns	Methodology	Ground truth	Benchmark samples
Alizadeh et al. 2020 [1]	Information operations in the U.S. by entities linked to China, Russia, Venezuela	4	Content-based approach	Accounts involved in information operations (released by Twitter and Reddit)	5,000 random U.S. users, 5,000 politically engaged U.S. users
Guarino et al. 2020 [2]	Constitutional referendum, Italy, 2016	1	Network-based approach	No ground truth	No ground truth
Gurajala et al. [3]	62 million users and their tweets crawled from Twitter	not specified	Pattern-matching on screen-names and tweeting times	No ground truth	Random sample of Twitter users (called “ground truth” in the paper)
Keller et al. 2019 [4]	Election campaign, South Korea, 2021	1	Network-based approach	Confiscated lists of accounts published in court proceedings	Regular users posting on election campaign

Table S1: Research design of related studies *(continued)*

Study	Data	Astroturfing campaigns	Methodology	Ground truth	Benchmark samples
Schoch et al. 2021 **(this paper)**	Information operations by state linked entities	35	Network-based approach	Accounts involved in information operations (released by Twitter)	Systematically constructed country-specific location-based random samples and hashtag-based random samples
Vargas et al. 2020 [5]	Information operations by state linked entities	10	Network-based approach + machine learning	Accounts involved in information operations (released by Twitter)	UK Parliament, US Congress, Academics, Random

S2 Overview of all astroturfing campaigns identified by Twitter

Table S2 summarizes all data releases by Twitter until mid-September 2020. The dataset identifiers are taken from the names of the files that Twitter provided. Note that some data sets actually are part of the same campaign according to Twitter, such as the three data sets on China released in 2019. The co-tweet analysis also indicates that the Iranian data sets released in 2019 are likely part of the same campaign. We also include data on South Korea, which was never discovered or released by Twitter, but which we use in our analysis.

Table S2 shows that the campaigns employed a variety of strategies. Some campaigns relied mostly on retweeting. Others made extensive use of hashtags in an attempt to get them to trend. Campaigns also differ in their propensity to link to outside material, i.e., how often they share newspaper articles or links to social contents like YouTube videos. The column % Detectable (accounts) indicates the percentage of accounts that would be classified as belonging to an astroturfing campaign based on our co-tweet or co-retweet method, meaning they either co-tweet or co-retweet with another account within a one minute time window.

Table S2: Statistics of all astroturfing campaigns identified by Twitter including number of tweets and accounts involved, percentage of retweets, tweets containing hashtags and URLs, and detectable accounts using co-(re)tweeting.

Campaign	Country	Tweets	Accounts	% RT	Tweets % #	% URLs	% Detectable accounts
armenia_202012	Armenia	72,960	35	0	98	98	62
bangladesh_201901_1	Bangladesh	26,212	15	4	7	90	0
catalonia_201906_1	Catalonia	9,489	129	64	18	36	91
china_052020	China	348,608	23,750	52	42	48	53
china_082019_1	China	1,898,108	744	19	20	35	52
china_082019_2	China	1,708,078	196	40	20	41	58
china_082019_3	China	10,241,545	4,301	23	20	33	83
cuba_082020	Cuba	4,802,243	526	70	69	42	94
ecuador_082019_1	Ecuador	700,240	1,019	83	33	35	63
egypt_022020	Egypt	7,935,329	2,541	51	40	55	90
egypt_uae_082019_1	Egypt/UAE	214,898	271	33	82	71	82
ghana_nigeria_202003	Ghana/Nigeria	39,964	70	41	32	41	43
honduras_022020	Honduras	1,165,019	3,104	53	31	35	95
indonesia_022020	Indonesia	2,700,296	795	16	28	30	83
ira_201810	IRA	8,768,633	3,608	38	29	61	78
ira_092020	IRA	1,368	5	36	17	77	50
ira_202012	IRA	68,914	31	32	39	59	25
iran_201810	Iran	1,122,936	770	21	30	84	63
iran_201901_1	Iran	4,671,959	2,311	55	49	48	92
iran_201906_1	Iran	1,302,012	503	39	37	71	61
iran_201906_2	Iran	1,963,141	238	82	41	35	65
iran_201906_3	Iran	254,781	2,861	35	38	35	39
iran_092020	Iran	2,451	104	23	16	51	96
iran_202012	Iran	560,571	238	18	43	65	20
qatar_082020	Qatar	220,254	33	74	26	24	74
russia_052020	Russia	3,434,792	1,153	12	11	59	76
russia_201901_1	Russia	920,761	416	77	43	60	66
russia_201906_1	Russia	3	3	0	0	67	0
russia_202012	Russia	26,684	70	14	49	79	28
sa_eg_ae_022020	Saudi Arabia/Egypt/UAE	36,523,980	5,350	91	41	42	78
saudi_arabia_082019_1	Saudi Arabia	340	6	9	25	91	0
saudi_arabia_112019	Saudi Arabia	32,054,257	5,929	50	28	37	73
serbia_022020	Serbia	43,067,074	8,558	85	15	46	67
spain_082019_1	Spain	56,712	259	48	62	29	94
thailand_092020	Thailand	21,385	926	52	20	23	58
turkey_052020	Turkey	36,948,536	7,340	75	24	33	69
uae_082019_1	UAE	1,325,530	4,248	49	53	49	88
venezuela_201901_1	Venezuela	8,950,562	1,196	24	46	79	85
venezuela_201901_2	Venezuela	984,980	755	7	13	96	86
venezuela_201906_1	Venezuela	569,453	33	5	2	99	88

Table S3 provides the same statistics for the datasets used in the main paper. The three IRA data sets targeting Germany, Russia and the U.S. were extracted from the complete IRA data set released by Twitter using the account languages German, English and Russian, respectively. The Hong Kong data set is a truncated version of the three China-related data sets released in 2019, limited to the time period relevant for the Hong Kong protests. It discards any tweets posted before April 1, 2019, as many of those accounts were apparently used for different (spamming) purposes before they became part of a more targeted, China-led campaign. The South Korea dataset is a 10% live collection of all Korean language tweets posted in June-December 2012.

Table S3: Statistics of astroturfing datasets analyzed in the main paper, including number of tweets and accounts involved, percentage of retweets, tweets containing hashtags and URLs, and detectable accounts using co-(re)tweeting.

					Tweets		% Detectable
Campaign	Country	Tweets	Accounts	% RT	% #	% URLs	accounts
germany_ira_201810	Germany	102,657	111	12	23	83	87
hong_kong_201904	Hong Kong	710,807	5,241	38	15	35	63
russia_ira_201810	Russia	3,953,675	1,039	40	18	59	93
south_korea_201212	South Korea	194,190	1,002	48	5	34	96
usa_ira_201810	USA	4,606,393	2,382	37	38	41	87

S3 Construction of random samples

Table S4: Statistics of random sample datasets, including number of tweets and accounts involved, percentage of retweets, tweets containing hashtags and URLs
.

Case	Country	Sample	Source	Sampling	Time period	Tweets	Accounts	% RT	Tweets % #	% URLs
germany_ira_201810	Germany (IRA)	Random	Brandwatch	Location-based		186,681	111	32	19	61
		Hashtag-based	Brandwatch	Top 30 hashtags	06/2017 - 08/2017	22,633	72	42	34	58
hong_kong_201904	Hong Kong	Random	Brandwatch	Location-based	04/2019 - 08/2019	867,953	4,524	48	30	50
		Hashtag-based	Brandwatch	Top 10 hashtags*	04/2019 - 08/2019	708,775	4,524	54	20	51
russia_ira_201810	Russia (IRA)	Random	Brandwatch	Location-based		4,009,148	1,012	30	17	53
		Hashtag-based	Brandwatch	Top 30 hashtags	06/2014 - 08/2014	390,596	530	35	21	52
south_korea_201212	South Korea	Random	10% Stream	Language-based		189,412	801	25	4	51
		Hashtag-based	10% Stream	Top 30 hashtags	06/2012 - 01/2013	194,188	813	41	8	54
usa_ira_201810	USA (IRA)	Random	Brandwatch	Location-based		5,367,473	2,645	33	18	54
		Hashtag-based	Brandwatch	Top 30 hashtags	03/2017 - 10/2017	544,536	455	58	34	62
venezuela_201901_1	Venezuela	Random	Brandwatch	Location-based		7,958,603	987	38	29	62
		Hashtag-based	Brandwatch	Top 30 hashtags	01/2015 - 12/2015	1,247,134	312	47	33	58

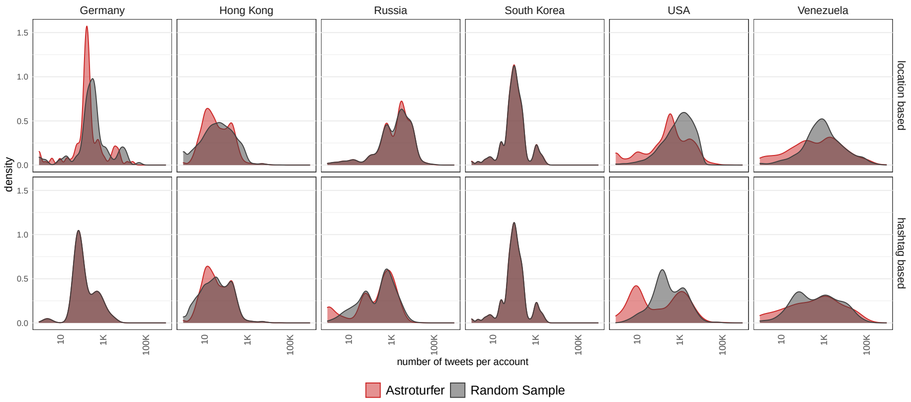

Figure S1: Matching of astroturfing accounts and random users, stratified by number of tweets.

S4 Analysis of all astroturfing campaigns identified by Twitter

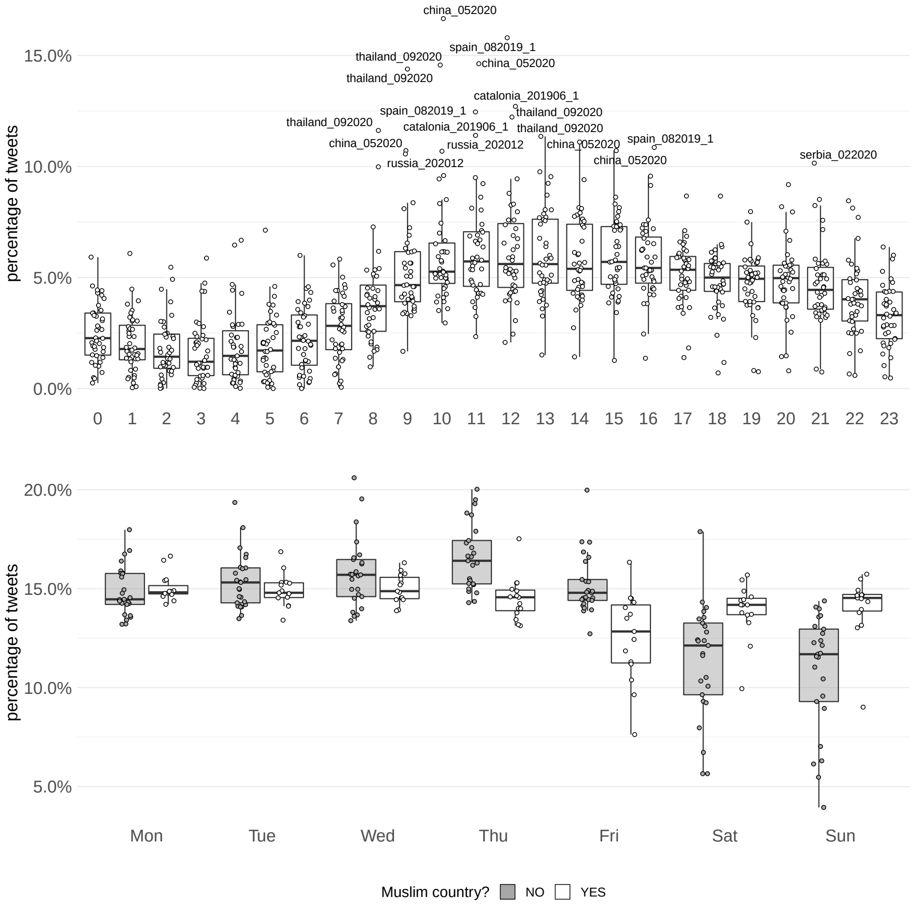

Figure S2: Percentage of tweets for all campaigns (top) per hour and (bottom) per weekday split by Muslim/non-Muslim countries. Excluding russia_201906_1 and saudi_arabia_082019_1 which had a miniscule number of tweets.

S5 Temporal patterns of astroturfing activity

Figure S3 replicates Figure 1 in the main paper, but for the hashtag-based instead of the location-based random samples.

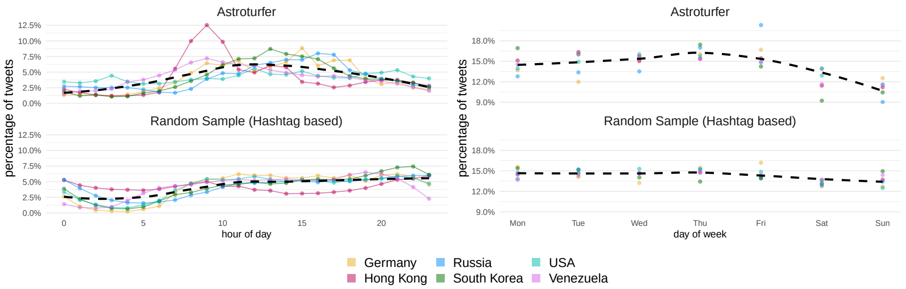

Figure S3: Comparison of hourly (left) and weekly (right) activity of astroturfing campaigns and random samples.

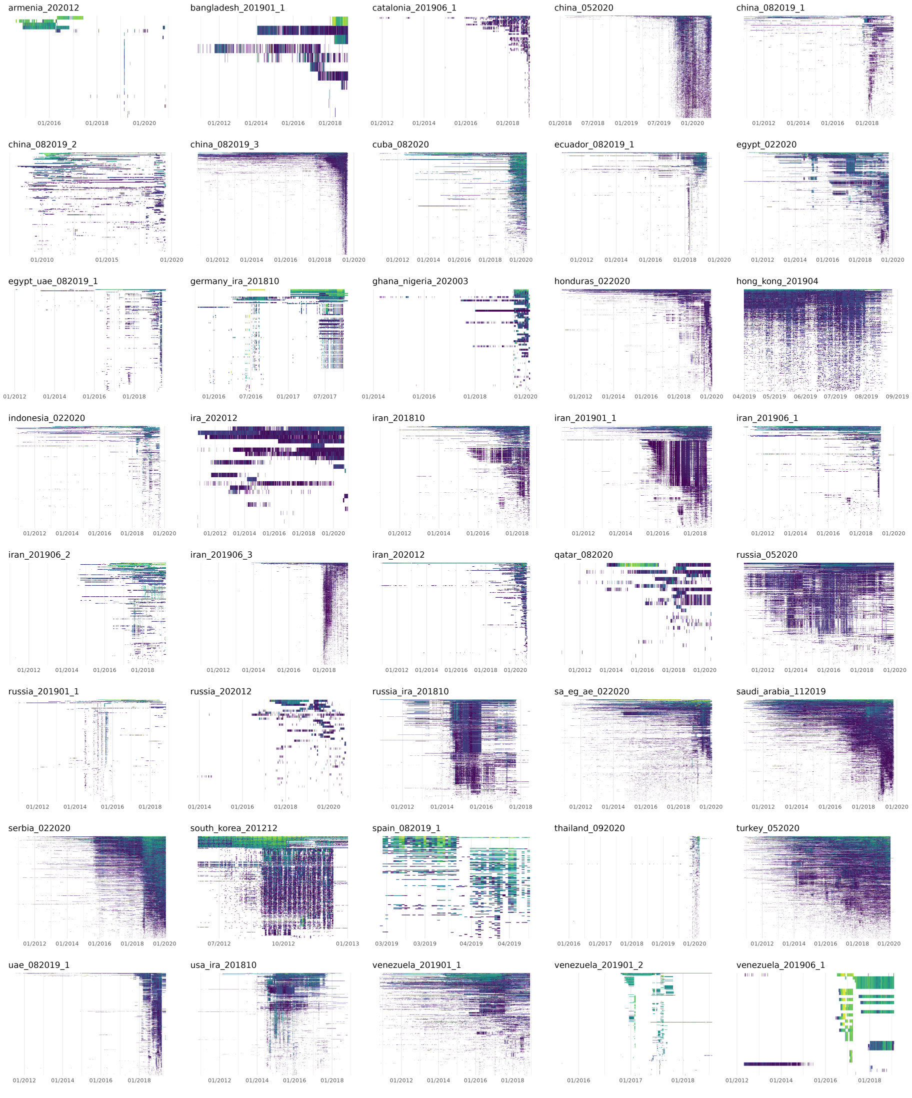

Figure S4: Daily activity pattern of astroturfing accounts, excluding russia_201906_1 and saudi_arabia_082019_1 which had a miniscule number of tweets.

S6 Full network figures and varying temporal thresholds

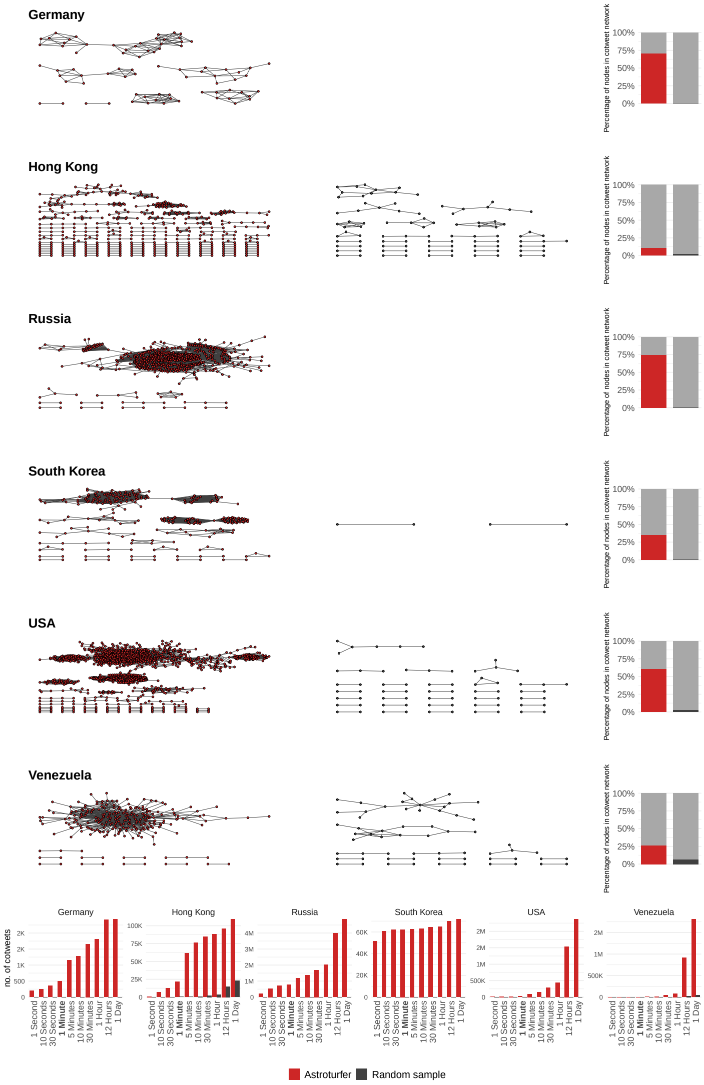

Figure S5: Comparison of co-tweets between astroturfing campaigns and location-based random samples.

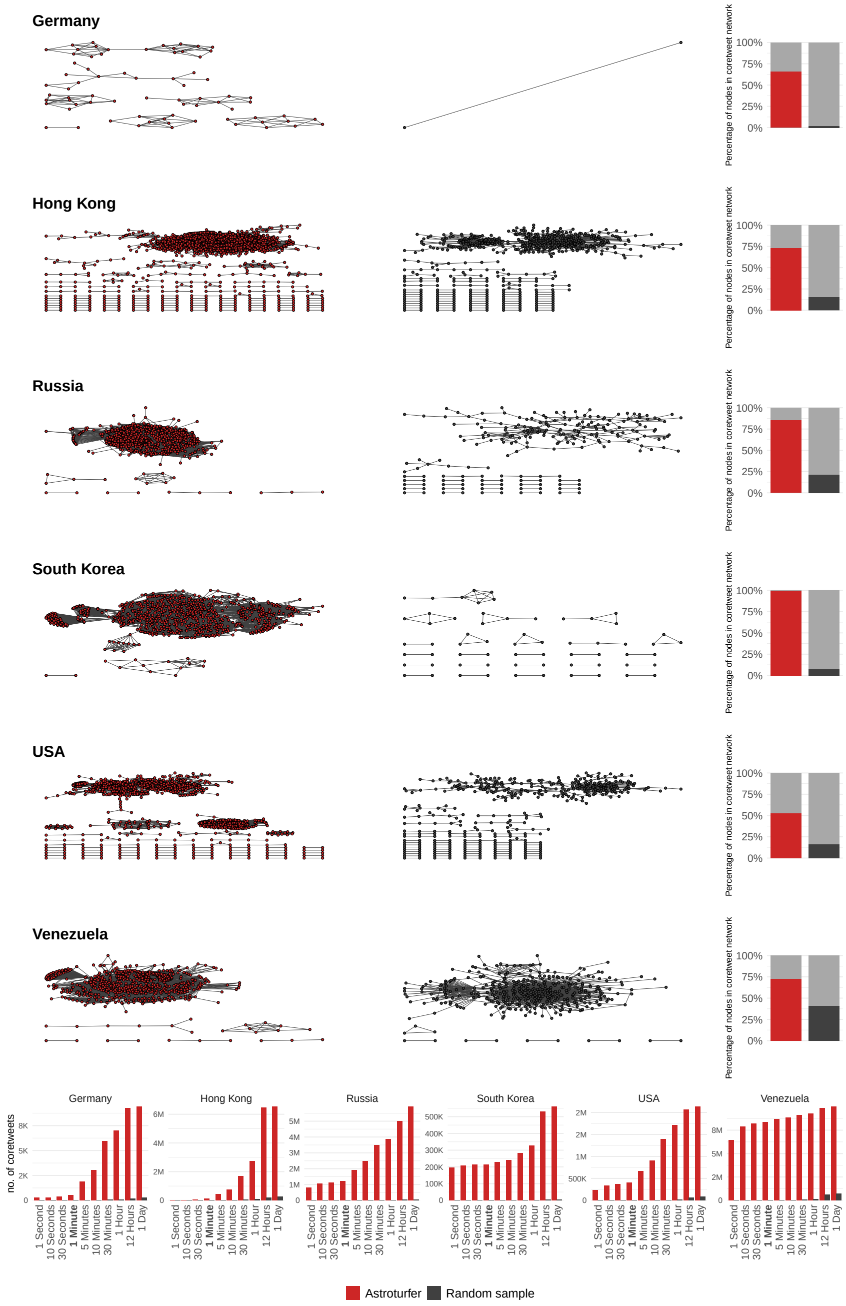

Figure S6: Comparison of co-retweets between astroturfing campaigns and location-based random samples.

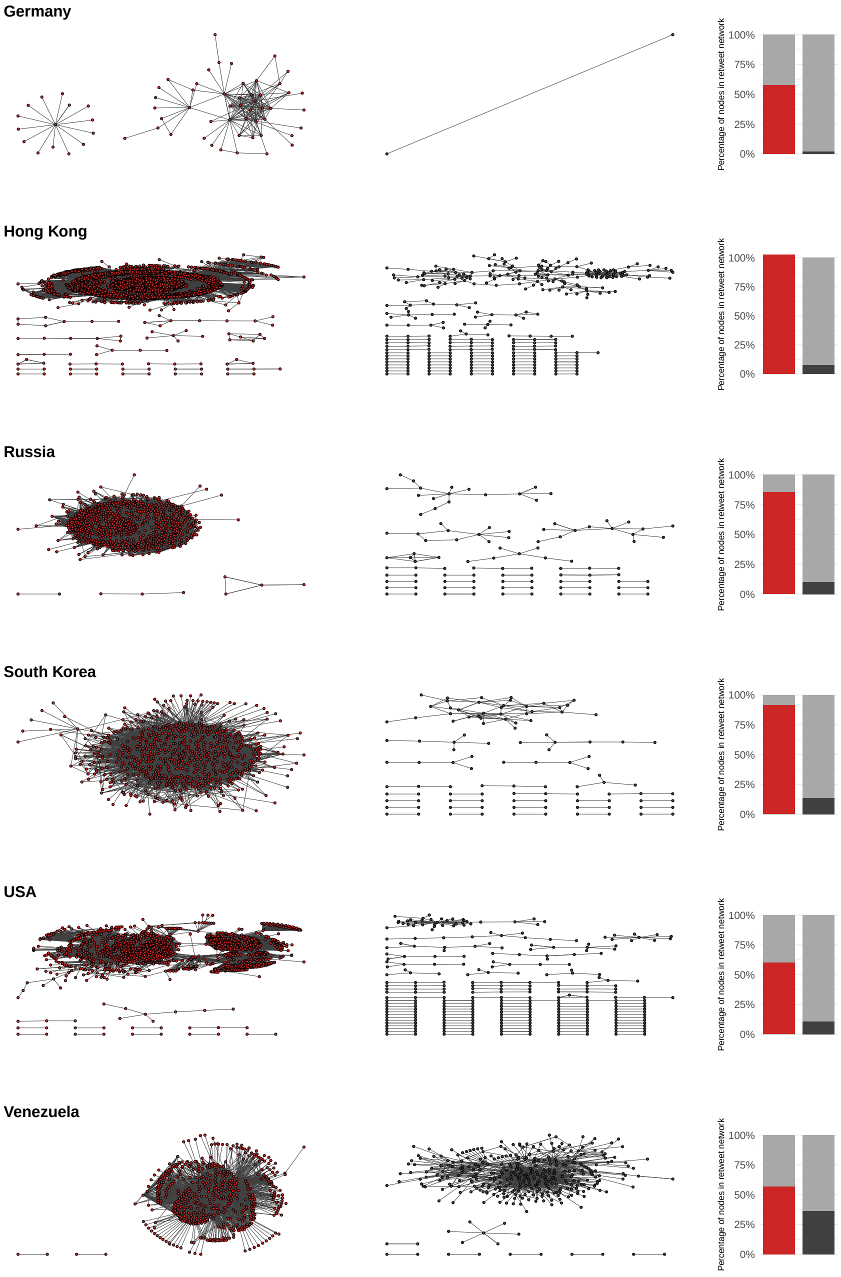

Figure S7: Comparison of retweets between astroturfing campaigns and location-based random samples.

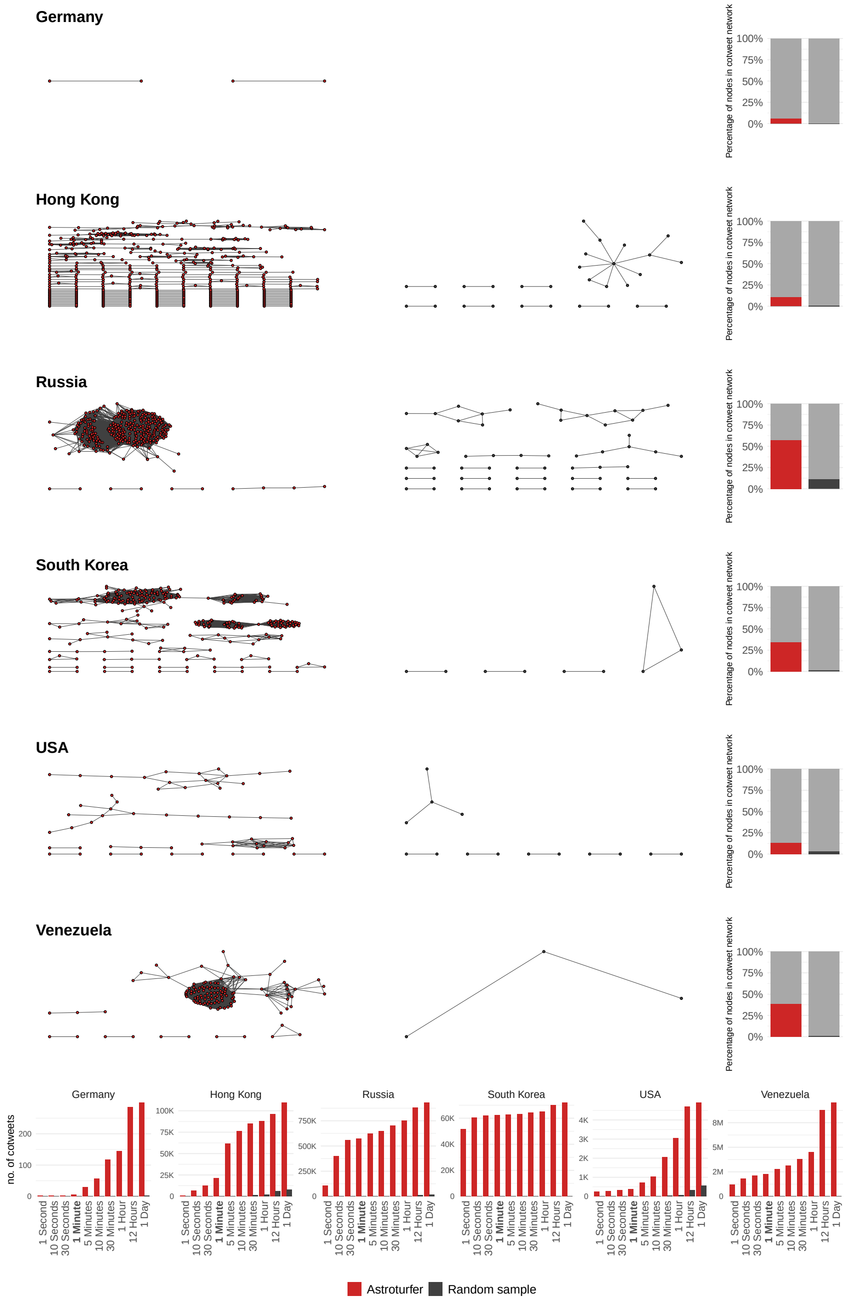

Figure S8: Comparison of co-tweets between astroturfing campaigns and hashtag-based random samples.

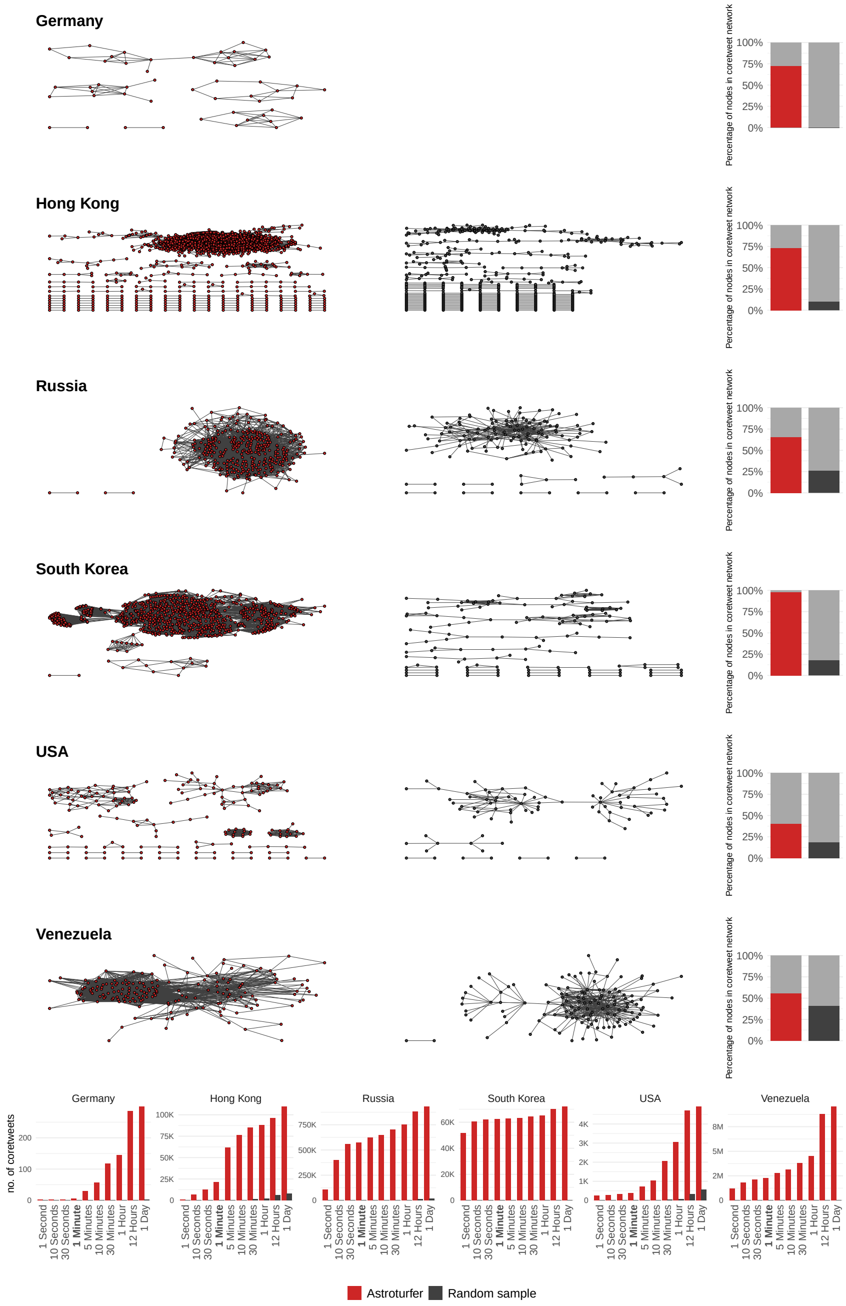

Figure S9: Comparison of co-retweets between astroturfing campaigns and hashtag-based random samples.

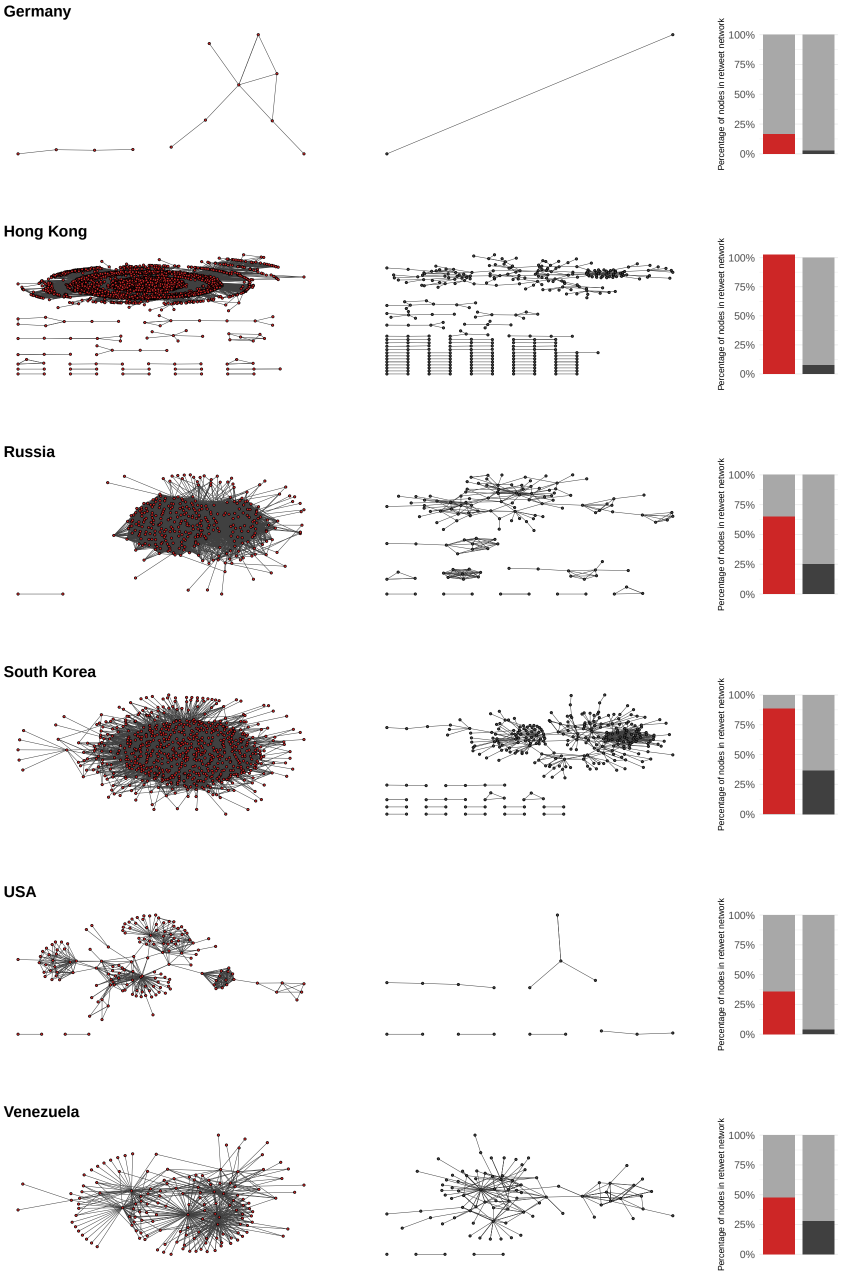

Figure S10: Comparison of retweets between astroturfing campaigns and hashtag-based random samples.

S7 #allesindenarm – a grassroots campaign as another benchmark

In the main analysis, we used two different random samples as our benchmark and have argued that those samples were constructed to resemble the kinds of accounts that the astroturfing campaigns try to imitate. However, such a random sample may not contain many accounts that are connected with each other and therefore may be unlikely to coordinate. In contrast, grassroots movements may be initiated by groups of accounts that are aware of each other and therefore may coordinate, even in the absence of centralized instructions. But retroactively finding a suitable comparable grassroots campaign for each of our astroturfing campaigns appears impossible: it would require an in-depth study of the respective Twittersphere in the relevant language and knowledge of the information campaigns active at the time.

In order to give the reader a sense of the type of message coordination occurring during a grassroots campaign and the associated network patterns, we therefore examine a German grassroots campaign to boost the vaccination campaign against COVID-19 taking place in November 2021. Under the hashtag #allesindenArm (roughly translated as "get a jab") influencers and regular social media users posted personal messages to motivate other users to get vaccinated. However, there was also fierce resistance on Twitter with people arguing against the campaign and vaccines in general (https://www.br.de/nachrichten/netzwelt/allesindenarm-was-steckt-hinter-dem-social-trend).

Our manual inspection of the tweets posted indicated that anti-vaxx accounts and users associated with the "Querdenken" movement that mobilizes against governmental COVID-19 countermeasures hijacked the hashtag to spread their critical message. Yet we have no evidence that those accounts engaged in astroturfing – and even if they did, it would only make the test of distinguishing this genuine grassroots campaign from astroturfing harder for us. This case can be regarded as a "hard" test in other regards as well, as several celebrities were part of the campaign, giving it somewhat more central coordination that one would usually expect in a pure grassroots campaign.

In order to collect the data on this case, we gathered the around 130,000 tweets that used the hashtag #allesindenArm starting from the beginning of 2021 using the Academic Research API (the vast majority of tweets were posted on November 14 and 15). We selected the accounts most active in the campaign, i.e., those 1,707 that posted at least five tweets using the hashtag, which gives us 10% of all the around 17,000 users that used the hashtag. This selection method again makes this a harder test: the more active a user, the more likely they are to post the same message as another user.

We then proceeded to gather the last 3200 tweets that these 1,707 accounts posted, and search for instances of co-tweeting or co-retweeting. Figure S11 shows the combined co-tweet and co-retweet network, with the same thresholds applied as in our astroturfing and comparison samples. Unlike in the case of all astroturfing campaigns, there is no large network component that contains the

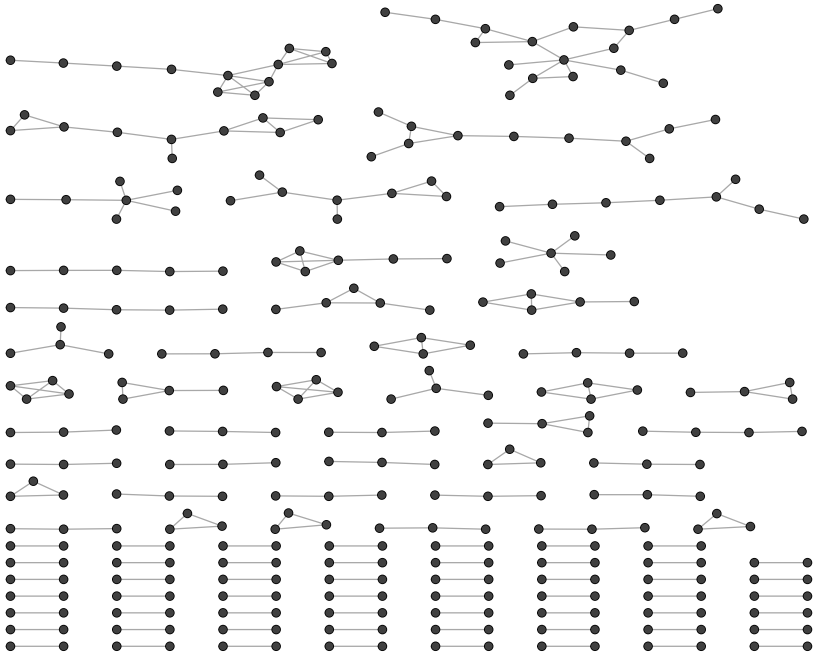

Figure S11: Message coordination among the 1,707 accounts most involved in the grassroots campaign #allesindenArm. Isolates not shown.

majority of the accounts involved. Only 320 of the 1707 accounts appear in this network that contains only 260 edges (and thus has a density of 0.005). The network more closely resembles that of the comparison samples: a few smaller components, with the vast majority of accounts (not shown) being isolates. In short: while there is some organic coordination of messages among the accounts participating in this grassroots campaign, it is much more limited than the (centralized) coordination we observe in all the astroturfing campaigns examined in this paper.

One might suspect that by selecting only 10% of the participants in this campaign – even if they are the most active ones – we are choosing a sample of unconnected users that cannot organically coordinate their messages because they aren't aware of each other – thus resulting in an underestimation of this type of message coordination in genuine grassroots campaigns. However, as Figure S12 demonstrates, a significant proportion of these 1,707 accounts (1624 nodes and 46298 edges (density 0.02) do indeed follow each other and could potentially co-tweet or copy-paste messages from each other, but do not nearly engage as frequently in these practices as astroturfing campaigns do.

While the #allesindenArm campaign is just one specific grassroots campaign, we take this as evidence that genuine grassroots campaigns do indeed

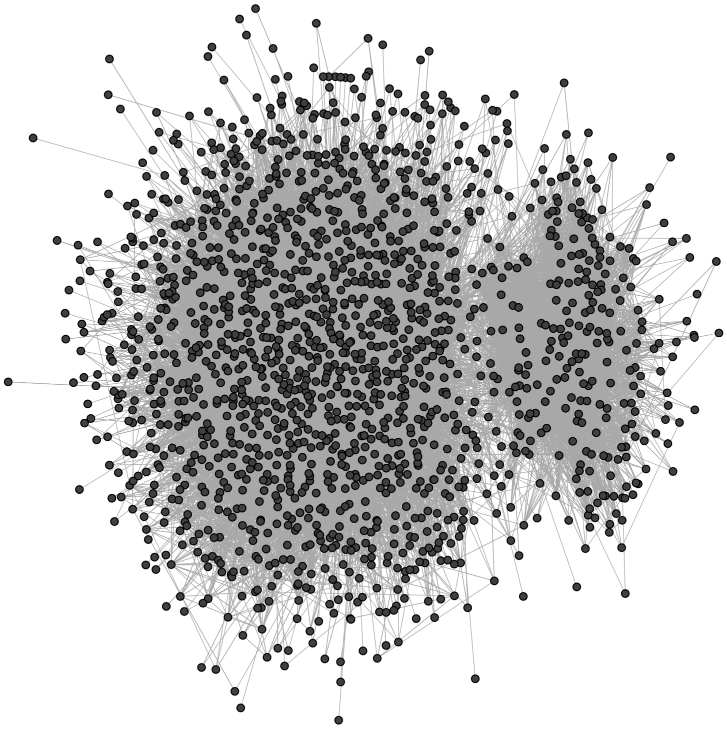

Figure S12: Follower network of the 1,707 accounts most involved in the grassroots campaign #allesindenArm.

display dissimilar message coordination patterns from astroturfing campaigns – in fact, they seem much more similar to the comparison samples presented in the main text. This also pertains to the hourly and day-of-week patterns, as Figures S13 and S14 show: participating accounts tend to be most active after work and on the weekends, and not during office hours/days.

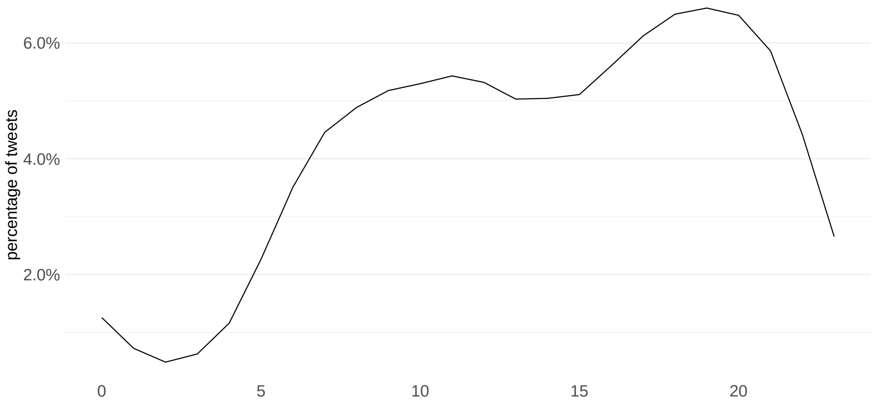

Figure S13: Hourly activity of the 1,707 accounts most involved in the grassroots campaign #allesindenArm.

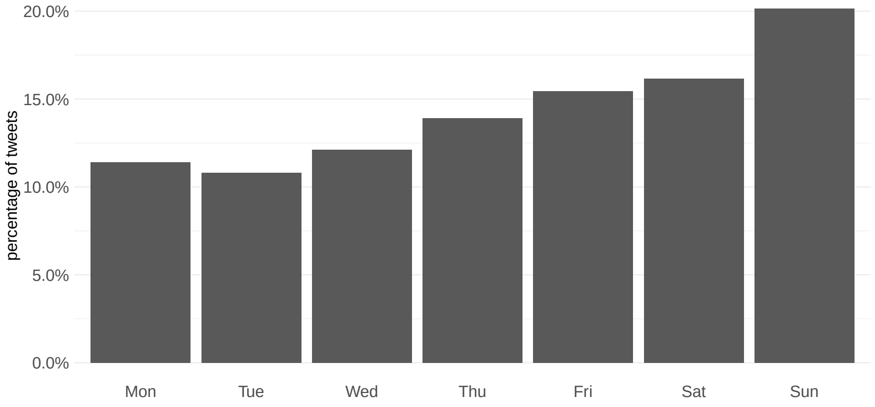

Figure S14: Activity by weekday of the 1,707 accounts most involved in the grassroots campaign #allesindenArm.

References

[1] Meysam Alizadeh, Jacob N. Shapiro, Cody Buntain, and Joshua A. Tucker. Content-based features predict social media influence operations. *Science Advances*, 6(30), 2020. doi: 10.1126/sciadv.abb5824.

[2] Stefano Guarino, Noemi Trino, Alessandro Celestini, Alessandro Chessa, and Gianni Riotta. Characterizing networks of propaganda on Twitter: a case study. *Applied Network Science*, 5(1), December 2020. doi: 10.1007/s41109-020-00286-y.

[3] Supraja Gurajala, Joshua S. White, Brian Hudson, and Jeanna N. Matthews. Fake Twitter accounts: profile characteristics obtained using an activity-based pattern detection approach. In *Proceedings of the 2015 International Conference on Social Media & Society - SMSociety '15*, Toronto, Ontario, Canada, 2015. ACM Press. doi: 10.1145/2789187.2789206.

[4] Franziska B. Keller, David Schoch, Sebastian Stier, and JungHwan Yang. Political Astroturfing on Twitter: How to coordinate a disinformation campaign. *Political Communication*, 37(2):256–280, 2020.

[5] Luis Vargas, Patrick Emami, and Patrick Traynor. On the detection of disinformation campaign activity with network analysis, 2020.
